# Convergences and divergences between scientific and Indigenous and Local Knowledge contribute to inform carnivore conservation

**DOI:** 10.1007/s13280-020-01443-4

**Published:** 2021-01-13

**Authors:** Miquel Torrents-Ticó, Álvaro Fernández-Llamazares, Daniel Burgas, Mar Cabeza

**Affiliations:** 1 Global Change and Conservation (GCC), Organismal and Evolutionary Biology Research Programme, Faculty of Biological and Environmental Sciences, University of Helsinki, P.O. Box 65, 00014 Helsinki, Finland; 2 Helsinki Institute of Sustainability Science (HELSUS), Faculty of Biological and Environmental Sciences, University of Helsinki, P.O. Box 4, 00014 Helsinki, Finland; 3 Department of Biological and Environmental Sciences, University of Jyväskylä, P.O. Box 35, 40014 Jyväskylä, Finland

**Keywords:** Camera trapping, Carnivore conservation, Indigenous and Local Knowledge, Scientific knowledge, Track survey

## Abstract

There is increasing recognition that diverse knowledge systems can work in mutually enriching ways and that Indigenous and Local Knowledge (ILK) can enhance biodiversity conservation. However, studies using scientific knowledge and ILK in a complementary manner, and acknowledging convergent and especially divergent insights have remained limited. In this study, we contrasted proxies of abundances and trends of threatened and conflict-prone carnivores (caracal, cheetah, jackal, lion, leopard, spotted hyaena, striped hyaena) derived separately from scientific knowledge and ILK. We conducted camera trapping, track surveys and semi-structured interviews with local pastoralists from northern Kenya. We found convergences highlighting the need for conservation action and divergences suggesting scientific ecological sampling limitations or underlying socio-psychological phenomena. Overall, our study shows that complementing scientific knowledge and ILK as separate sources of information and opening up space for discrepancies can enrich our understanding of the status and trends of carnivores, as well as recognizing human-carnivore relationships.

**Electronic supplementary material:**

The online version of this article (10.1007/s13280-020-01443-4) contains supplementary material, which is available to authorized users.

## Introduction

There is increasing recognition, both in academic and policy circles, that complementing different knowledge systems is key to widen the evidence basis underpinning wildlife management and biodiversity conservation (Whyte et al. 2016; Kutz and Tomaselli 2019; Hill et al. 2020). In fact, the idea that diverse knowledge systems can work in mutually enriching ways is well reflected in the aspirations of the Intergovernmental Science-Policy Platform on Biodiversity and Ecosystem Services (IPBES) and the Convention on Biological Diversity, both of which have explicitly emphasized that Indigenous and Local Knowledge (hereinafter ILK) can contribute to conservation, policy and practice (IPBES 2019).

Along these lines, much research has contrasted different knowledge systems to further our understanding of different social-ecological processes, from local to global scales (e.g., Fernández-Llamazares et al. 2017; Morales-Reyes et al. 2019). For instance, research has tapped into ILK to expand and deepen our knowledge of the local impacts of climate change (e.g., Fernández-Llamazares et al. 2017), land-use changes and habitat degradation (e.g., Admasu et al. 2010), wildlife health (e.g., Kutz and Tomaselli 2019), or nature's contributions to people (e.g., García-Alfonso et al. 2019), to cite just a few. However, despite this growing body of literature, there is still a ubiquitous tendency to validate ILK with scientific knowledge (see Tengö et al. 2014 for a discussion). Many researchers have warned against such external scientific validations, arguing that they can lead to the dismissal of valid and useful knowledge (Bohensky and Maru 2011; Díaz et al. 2015; Tengö et al. 2017; Hill et al. 2020) and to the disempowerment of ILK holders (Roué and Nakashima 2018). To avoid this, it has often been suggested that ILK and scientific knowledge should be used as complementary in frameworks that enable synergies between knowledge systems (Tengö et al. 2014; Whyte et al. 2016; Kutz and Tomaselli 2019), opening up space for diverse, and often diverging, insights and perspectives (Tengö et al. 2014, 2017).

Most ecological studies addressing ILK have often focused on gathering ecosystem-level information (e.g., Admasu et al. 2010; Kutz and Tomaselli 2019). Yet, there is a significant number of ecological studies where ILK is being progressively explored also at the species level, especially in marine ecology (e.g., Butler et al. 2012). Fewer studies have incorporated ILK in terrestrial ecology estimating species abundances (e.g., Anadón et al. 2009) and population trends (e.g. Kamgaing et al. 2019), both of which are crucial for both conservation and wildlife management. Notably, many studies on ILK at the species level have focused on relatively highly abundant and easily detectable species, and/or species with significant socio-economic or cultural importance for Indigenous Peoples and Local Communities (e.g., Fernández-Llamazares et al. 2016). Much less research has looked at ILK in relation to scarce, cryptic, elusive or nocturnal species (e.g., Reibelt et al. 2017), or species that might be considered as conflict-prone in certain cultural and socio-economic contexts (e.g., Kuriyan 2002), where ILK may be most relevant. Several carnivore species fit all these criteria, and not surprisingly, research on this species group from an ILK perspective has been more limited, particularly where threatened carnivores coexist with local communities (but see Padmanaba et al. 2013 or Sahoo et al. 2013 for some examples).

Many carnivore species are keystone species, and their loss may have cascading effects on communities and ecosystems (Ripple et al. 2014). Increasing conflicts with humans (Ripple et al. 2014) and habitat loss (Winterbach et al. 2013) have resulted in many carnivores being listed among the most threatened species globally (Wolf and Ripple 2017). Robust and legitimate knowledge of carnivore abundances and their population trends is paramount for the effective conservation management of such carnivores (Gese 2001).

Historically, carnivore conservation initiatives have been based on scientific knowledge alone, drawing from a diverse range of sampling methods, such as camera trapping and track (spoor) survey (see Wilson and Delahay 2001; de Iongh et al. 2011; Pirie et al. 2016). Nevertheless, estimates from commonly used sampling methods can be rather uncertain, with results varying substantially between methods (e.g., Torrents-Ticó et al. 2017). Low abundances and decreasing trends of most carnivores call for intensive sampling efforts, and here is where the contributions of ILK holders can play a critical role. First, ILK can fill research gaps in areas where scientific data on carnivores are meagre at best (e.g., Padmanaba et al. 2013; Sahoo et al. 2013). Second, collaborations between Indigenous Peoples and researchers can further our understanding of several species ecological distribution ranges, baselines and trends (Skroblin et al. 2019), as well as recognizing local perceptions, attitudes and values towards these species. However, despite the positive contributions of ILK, studies rarely link ILK to scientific knowledge, and when they do, ILK reliability is assessed with scientific knowledge (see Gandiwa 2012 or Caruso et al. 2017 for some examples). Few studies have taken advantage of an approach that looks at the two knowledge systems as complementary (but see Dolrenry et al. 2016 for an exception), which can be key not only to understand abundances and trends, but also to recognize the needs and challenges for conservation. A prominent example is the Lion Guardians program in the Amboseli Ecosystem (southern Kenya), which provides an interesting reflection on how complementing conventional scientific monitoring with ILK can help to monitor lion movements in a participatory way (Dolrenry et al. 2016).

In this study, we complement information and insights derived from two different knowledge systems (i.e., ILK and scientific knowledge) to: (a) obtain an enriched picture of understanding of the status and trends of scarce, elusive and conflict-prone carnivore species; and (b) reflect on approaches and procedures to work across independent knowledge systems for enhanced carnivore conservation. To meet these purposes, we contrast proxies of abundances and trends of carnivore species, derived separately from common scientific sampling methods and semi-structured interviews among a pastoralist community from northern Kenya. We then discuss though out a qualitative approach on how these information sources converge or diverge and elaborate on their value in informing carnivore conservation.

## Theoretical background

In this article, we draw on IPBES Conceptual Framework that embraces different knowledge systems or “agents, practices and institutions that organize the production, transfer and use of knowledge” (McElwee et al. 2020). On the one hand, we use the term ILK that is widely used in science-policy circles (e.g., IPBES 2019), and is defined as “knowledge and know‐how accumulated across generations, which guide human societies in their innumerable interactions with their surrounding environment” (McElwee et al. 2020). Yet, we acknowledge that what can be considered as knowledge is still heatedly debated (Raymond et al. 2010), for instance, the local experience with one’s surroundings can be considered as knowledge or perception (see Yeh 2016 for a discussion). The literature has to date interchangeably used the terms “knowledge” (e.g., Anadón et al. 2009; Kamgaing et al. 2019) and “perception” (e.g., Leong 2009) to refer to local reports of species abundances and trends. For the purpose of this study, it is important to note that we consider observations as being only one dimension of a larger system of locally-developed knowledge (see Orlove et al. 2010). Such observations are constructed and appraised based on a wider context of interpretation and evaluation of culturally relevant information from multiple sources (Berkes et al. 2000). In general, observing elusive carnivore species requires very specific and place-based knowledge, much of which is cumulative and socially transmitted. Local observations are, therefore, anchored on a larger cultural context and encoded on the cultural performance of everyday activities, local speech and other time-honoured cultural traditions passed from generation to generation (see Reyes-García and Fernández-Llamazares 2019). Local observations should be thus seen as a form of tacit and situated knowledge, reflecting a depth of embodied experience and with an inherent intergenerational dimension (see Fernández-Llamazares et al. 2016).

On the other hand, we use the term scientific knowledge to refer to “knowledge typically generated in universities, research institutions and private firms following paradigms and methods typically associated with the scientific method” (Díaz et al. 2015). In this study, scientific knowledge refers to the information obtained from two ecological sampling methods (i.e., track survey and camera trapping), and ILK refers to observations of wildlife by local Daasanach people, gathered through surveys and classic ethnographic methods (see Morales-Reyes et al. 2019 for an example using both terms ILK and scientific knowledge). These definitions reflect a partial and context-specific understanding of both knowledge systems.

We acknowledge ILK and scientific knowledge are not necessarily mutually exclusive (Díaz et al. 2015) and there is a variety of approaches to integrate knowledge systems (Raymond et al. 2010). Some studies have performed correlation and multivariate regression statistics (e.g., Anadón et al. 2009; Fernández-Llamazares et al. 2017), others have calculated the chance‐corrected percent agreement and the quantity disagreement statistics of Pontius (e.g., Aswani and Lauer 2014), and Morales-Reyes et al. (2019) have combined a mixed approach including non-parametric comparison tests, correlations, and covariance analyses. Here, we are interested in complementing scientific knowledge and ILK with a qualitative approach to look at convergences and divergences, which are normally dismissed, and show that all together can further and deepen our holistic knowledge of carnivores. We highlight the importance of keeping both knowledge systems as separate sources of information with open space for divergences, thereby making carnivore conservation more inclusive and socially legitimate.

## Materials and methods

### Study area and ethnic group

**Fig. 1:**
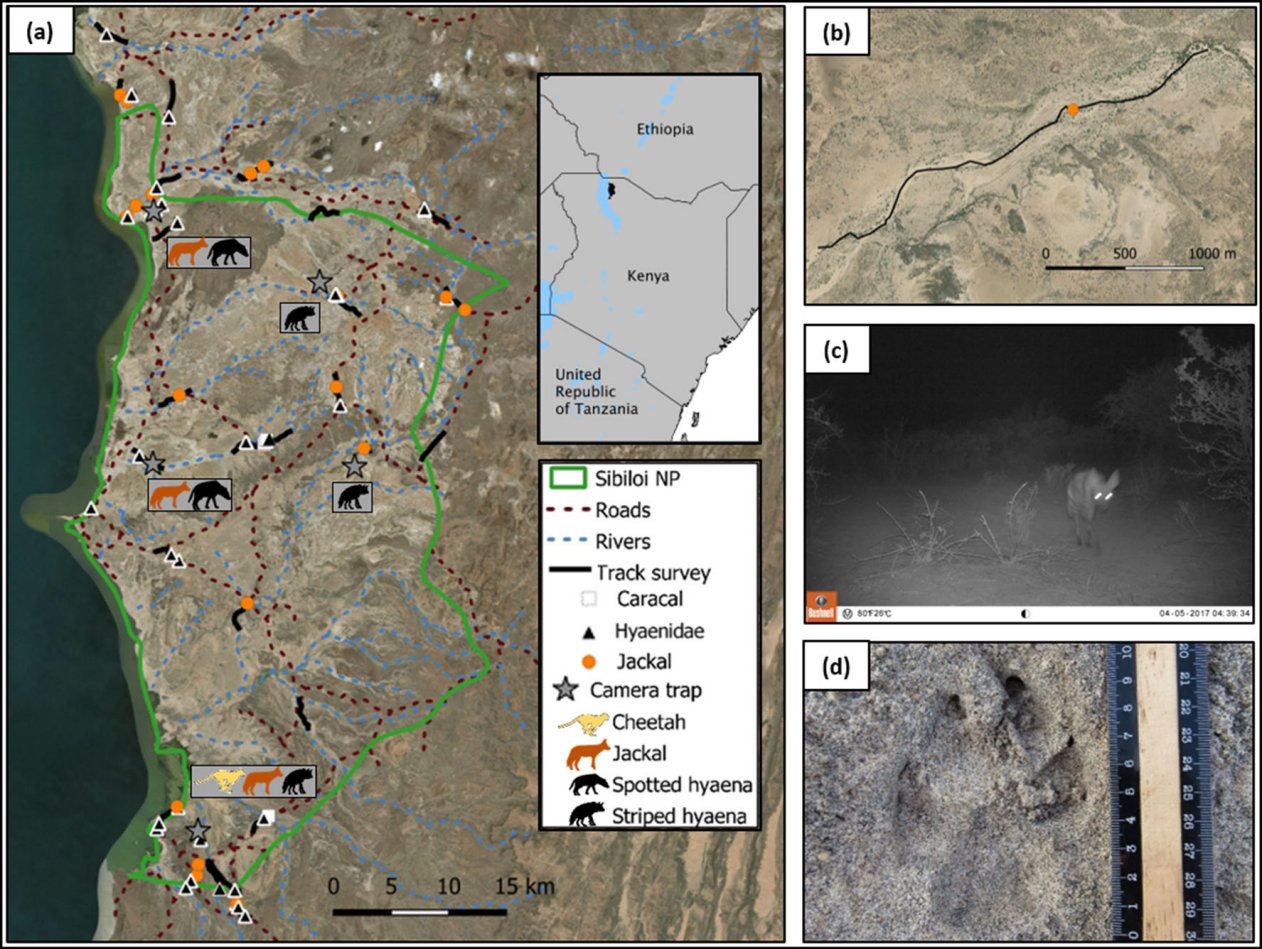
**a** Map of Sibiloi and surroundings presenting the spatial distribution of the species identified by camera trapping and track surveys. Geometric figures show the identified tracks by species (Caracal: white square, Hyaenidae species: black triangle, Jackal: orange circle). Stars mark the five areas where camera traps were deployed and the silhouettes illustrate the species identified by camera-trap photographs (cheetah: yellow, jackal: orange, spotted hyaena and striped hyaena: black). **b** Detailed example of the geography of the area and a track survey carried out along a riverbed; **c** striped hyaena photographed by a camera trap; and **d** Hyaena track

We conducted the study in the area of Sibiloi National Park (hereinafter Sibiloi) and its surroundings. Sibiloi has an extension of 1570 km^2^ and is located on the remote north-eastern shore of Lake Turkana, North Kenya (Fig. 1a). Sibiloi was established in 1973 and ecological studies have been scant and little is known about the carnivore species potentially found in the area (Table 1): caracal (*Caracal caracal*), cheetah (*Acinonyx jubatus*), leopard (*Panthera pardus*), lion (*Panthera leo*), spotted hyaena (*Crocuta crocuta*), striped hyaena (*Hyaena hyaena*) and two species of jackal, African golden wolf (*Canis anthus* or *Canis lupaster*) and black-backed jackal (*Canis mesomelas*). Sibiloi is located within the traditional territory of the Daasanach people that are largely considered as an agro-pastoral society (Almagor 1978), mostly herding cattle, sheep and goats. Subsistence hunting and fishing are also relatively common among the Daasanach, particularly under circumstances of dire famine. Wildlife holds strong sociocultural values among the Daasanach community, with a rich tradition of folktales, stories and songs about wildlife (see Daasanach community 2019 for some examples). Some Daasanach traditional ceremonies (e.g., Dimi) feature several customary uses of wildlife (e.g., cheetah and leopard skins; Mwamidi et al. 2018).

**Table 1:** Carnivore species potentially found in Sibiloi with English, Daasanach and scientific names. Global IUCN Status (LC: Least Concern; NT: Near Threatened; VU: Vulnerable) and Population trend obtained from www.iucnredlist.org. Species-specific traits: body mass, activity period and population density (following panTHERIA, Jones et al. 2009), social organization (following Dalerum 2007), and size of prey [following Gittleman 1985: Very small (< 1 kg), Small (1–10 kg), Medium (10–100 kg), Large (100–400 kg)]

Species			IUCN		Traits				
English name	Daasanach name	Scientific name	Status	Population trend	Body mass (kg)	Activity period	Population density (number/m^2^)	Social organization	Size of prey
Caracal	Kasaante'	*Caracal caracal*	LC	Unknown	12	Nocturnal	–	Flexible	Very small
Cheetah	Gosoch	*Acinonyx jubatus*	VU	Decreasing	51	Diurnal	0.01	Solitary	Medium
Jackal*	Baich	*Canis anthus*	LC	Decreasing	10	Nocturnal-Crepuscular	0.23	Flexible	Very small
		*Canis mesomelas*		Stable	8	Nocturnal-Crepuscular	0.74	Flexible	Small
Leopard	Mo'r dhatka'	*Panthera pardus*	VU	Decreasing	52	Nocturnal-Crepuscular	0.07	Flexible	Medium
Lion	Luoch	*Panthera leo*	VU	Decreasing	159	Nocturnal	0.11	Group	Large
Spotted hyaena	Lool	*Crocuta crocuta*	LC	Decreasing	63	Nocturnal	0.12	Group	Large
Striped hyaena	Na'gera	*Hyaena hyaena*	NT	Decreasing	35	Nocturnal-Crepuscular	–	Flexible	Very small**

*Jackal includes African golden wolf/Black-backed jackal (see “Materials and methods” section)

**Primarily scavenger on carcasses of large vertebrates, supplemented by hunting small vertebrates

This research was carried out with the authorization of Kenya Wildlife Service (KWS/BRM/5001) and the National Commission for Science, Technology and Innovation (NACOSTI/P/18/21446/20296).

### Scientific knowledge

We carried out camera trapping and track (spoor) surveys to assess the status of carnivore species, as they are the most common ecological sampling methods used for rapid and broad-scale assessments of wildlife abundance such as ours. Both methods were conducted in challenging logistical conditions of remote areas. The sampling was conditioned by accessibility (road distribution) and geology (track visibility). Despite these constraints, both ecological sampling methods were stratified and conducted side by side with a multi-taxa study, covering the major habitat types characterizing the landscape (i.e., riverine beds with tall trees, coastal grasslands and bushlands; see Fig. 1a).

*Camera trapping*. We deployed fifteen Bushnell Trophy Cam infra-red camera traps for a total of 300 camera-trapping nights during two different periods (November–December 2016 and March–April 2017). To increase the probability of photographing wildlife, especially our target carnivore species, we placed the camera traps along pathways in five locations (Fig. 1a) at a height of approximately 90‐100 cm. (Broekhuis et al. 2018). We programmed the camera traps to take three photos when triggered. We classified all photographs according to species, but we used only photographs of the studied carnivore species (see Fig. 1c for an example). We considered consecutive photographs as an ‘independent event’ when photographs were at least 60 min apart from the previous images of the same species at the same camera trap (Broekhuis et al. 2018).

*Track (spoor) survey*. We used a grid with a cell size of 10 × 10 km. Within each cell, the first author and a Daasanach track expert conducted 3 km transects during two periods (March–April 2017 and February–March 2018). We carried out track surveys on roads and dry riverbeds (Fig. 1b) because sandy substrates are suitable for track identification and frequently used by carnivores (Wilson and Delahay 2001). Some cells were not accessible, thus we allowed more flexibility in choosing transects (i.e., selecting closer transects from the nearby grid if surveying the selected cell was not feasible). We began the track surveys at sunrise because the low angle of the sun produces shadows in the tracks that facilitate their detection and identification (Pirie et al. 2016). We inspected each track found with a millimetre ruler and track identification guides (Stuart and Stuart 1998; Gutteridge and Liebenberg 2013) to ensure correct species identification. In addition, we photographed each carnivore track found with a ruler next to it (see Fig. 1d for an example). Photographs helped us to validate field identification. When a track was ambiguous in the field and the picture identification was still uncertain, we discarded the track from the analyses. Because we could not distinguish with confidence the highly similar tracks of spotted hyaena and striped hyaena (de Iongh et al. 2011), we grouped all those tracks as 'hyaenidae species' (both species are members of the family hyaenidae). We recorded all the identified track locations with a handheld GPS device (Fig. 1a).

### Indigenous and Local Knowledge (ILK)

A team composed of international researchers and Daasanach community members carried out 80 face-to-face semi-structured interviews during November and December 2016. Interviews have been widely used to obtain information on species abundances and population trends (see Anadón et al. 2009; Gandiwa 2012 for some examples). Semi-structured interviews are informal conversations where several questions are prepared in advance to obtain information on a specific subject. Interviews were carried out in Daasanach language and translated to English with local pastoralists that were found opportunistically when they were herding their livestock in the field. The study focused specifically on men because, in the Daasanach community, they have traditionally held the role of herding (Willnerd 2018). They herd the livestock in search of pastures over long distances and for several months visiting different areas of the park and surroundings in different seasons.

During the scoping phase of the project (February 2016), we also obtained permission from the Ileret Ward (the main administrative authority in the area) and the Daasanach Council of Elders (the main local customary institution representing the Daasanach people of North Kenya) to develop this project in collaboration with the local Daasanach community. The research design of this study is in accordance with the guidelines of the Ethical Review Board in the Humanities and Social and Behavioural Sciences of the University of Helsinki. Additionally, this research adhered to the Code of Ethics of the International Society of Ethnobiology. We obtained Free, Prior and Informed Consent (FPIC) from each ILK holder interviewed in the study, and we guaranteed ILK holders' anonymity, confidentiality and data protection throughout the entire study. All ILK holders agreed to be interviewed under these conditions.

During the initial testing phase, we ensured mutual comprehension of our semi-structured interview by looking for appropriate terms (e.g., species, abundance, sightings) that were easily understood by ILK holders, to enable mutual comprehension. We also evaluated the carnivore identification of ILK holders by using colour photographs of different species, including similar species that were not the focus of this study [e.g., African civet (*Civettictis civetta*), common genet (*Genetta genetta*)] in order to test possible misidentifications. For instance, local people did not distinguish between the African golden wolf and the black-backed jackal, and therefore, we grouped them as 'jackal' ('baich' in Daasanach language). Interviews were then carried out including the different species previously tested and clearly identified by the ILK holders. Only in very few cases there was confusion, and we did not continue the interview until it was clarified.

The semi-structured interview (see Table S1 for a sample of the protocol used) focused on: (a) the current abundance of carnivores through the local observation frequency; (b) the abundance of carnivores in the ILK holder's childhood; and (c) the last time each carnivore species was seen or heard (i.e., last sighting). We took detailed field notes during the interviews, recording perspectives and observations made by local pastoralists. We defined childhood age as the decade after birth, following Fernández-Llamazares et al. (2017). ILK holders normally indicated that they “did not know" if they could not answer a question.

### Data analyses

#### Scientific knowledge

We calculated photographic and track rates from camera trapping and track surveys, respectively. We defined photographic rate as the ratio of independent photographs to the number of trap days (number of 24-h periods during which cameras were operating). We defined track rate as the number of independent tracks to the total number of kilometres surveyed. We consider a relationship between the frequency of tracks or photographs and the relative index of abundance (Wilson and Delahay 2001), and thus, that higher photographic and track rates, the more abundant the species was. Yet, we acknowledge some limitations and biases in our approach. We could not distinguish between individuals through camera trapping and track surveys, and thus in some occasions the same individual may have been counted more than once. Consequently, our rates may reflect both the number and behaviour of animals. There are other factors that may influence index rates and were not considered in our approach (see Burton et al. 2015; Sollmann 2018). For instance ecological traits of species (see Table 1) may be linked to their probability of detection and therefore bias the relative differences between species (Burton et al. 2015). We recognize that our rates can be also understood as activity rates instead of abundance rates, where species activity at a specific place can increase either because individuals use that place more often or because more individuals use that place (Sollmann 2018). Because the frequencies we detected are so low, discerning between activity and abundance is less important. This is further supported by the fact that our results from camera traps and track surveys present similar patterns. In addition, because they are not very different from other studies using those methods in other parts of Africa with similar carnivore species composition (see Tables S2, S3). Thus, we believe that the rates provided suffice as proxies of carnivore abundances.

#### Indigenous and Local Knowledge (ILK)

We coded the current abundance and the abundance in the ILK holders' childhood as common = 2 (i.e., many individuals seen often); present in low numbers = 1 (i.e., some seen occasionally); absent = 0 (i.e., not seen) for each carnivore species. We grouped last sighting reports into three groups: 'This month', 'This year' and 'Over a year'. We calculated the descriptive statistics based on the number of ILK holders' answers to each question and we represented them by percentages. We acknowledge that, in some circumstances, local reports may include misidentifications (e.g., observations made at night in bushy areas from faraway) and that local pastoralists cannot always distinguish individuals of the same species, thus in some occasions the same individual may be counted more than once. That being said, we used reports of the current abundance and the last sighting of a given carnivore species as proxies for the carnivore abundance according to ILK. We considered that the higher the percentage of ILK holders that reported the current abundance as "common" and the more recent the last sighting for a given species were, the more abundant the species was in the area according to ILK holders. We excluded the "did not know" answer from the analyses. We calculated the carnivore population trend according to ILK as the difference between the current abundance and the abundance in ILK holder' childhood. We coded the population trend as − 1 = decrease, 0 = stable and 1 = increase. In this case, a decreased population trend indicates that the current abundance reported was lower than the abundance reported in ILK holder' childhood. The trend index average according to ILK (hereinafter Trend Index Average) was the average of the population trend among ILK holders for each carnivore species.

### Contrasting knowledge systems

We contrasted proxies of abundances and trends of carnivore species, obtained separately from scientific knowledge and ILK. Following these lines, we acknowledge that the distant timeline to which ecological sampling methods and semi-structured interviews were carried out may influence the points of convergence and divergence between both knowledge systems. For current abundances, we contrasted categorical percentages obtained from semi-structured interviews for ILK to photographic and track rates obtained through camera trapping and track surveys respectively (Table 2). For population trends, we contrasted the trend index average direction (positive or negative) acquired from semi-structured interviews for ILK to information gathered from a systematic review (Table 3). To the best of our knowledge, no research has studied carnivores’ population trends in Sibiloi. Nevertheless, we carried out a systematic review of the literature using different search engines (e.g., ISI, PubMed, Scholar). However, as the publications for Sibiloi are scant and often in non-indexed journals, only Scholar provided the few existing citations referring to our study species in Sibiloi. We paired the term "Sibiloi" with each of the carnivore species: "caracal", "cheetah", "jackal", "leopard", "lion", “spotted hyaena” and “striped hyaena”. Overall the search yielded a list of 219 results, out of which very few were relevant to our study (see Dolrenry et al. 2014; Cabeza et al. 2016a, b; IUCN 2017; Willnerd 2018).

**Table 2:** Summary of photographic rates (records per day) and track rates (records per km) for scientific knowledge, and for ILK, percentages of (a) current local abundances according to ILK; and (b) last sightings by ILK holders

Species	Scientific knowledge		Indigenous and Local Knowledge (ILK)							
			(a) Is the current abundance of this animal Absent/Present/Common?				(b) When was the last time that you remember seeing this animal?			
	Photographic rate	Track rate	*N*	Absent (0)	Present (1)	Common (2)	*N*	‘Over a year’	‘This year’	‘This month’
				(%)	(%)	(%)		(%)	(%)	(%)
Caracal	–	0.07	80	2.50	3.75	93.75	78	5.13	23.08	71.79
Cheetah	0.003	–	80	12.50	28.75	58.75	71	32.39	40.85	26.76
Jackal	0.017	0.18	80	0.00	6.25	93.75	80	1.25	8.75	90.00
Leopard	–	–	79	1.27	39.24	59.49	79	27.85	46.84	25.32
Lion	–	–	80	3.75	76.25	20.00	79	53.16	36.71	10.13
Spotted hyaena	0.01	0.33*	80	3.75	3.75	92.50	77	3.90	16.88	79.22
Striped hyaena	0.017		75	0.00	9.33	90.67	76	6.58	17.11	76.32

*Hyaenidae species (including spotted hyaena and striped hyaena, see methods). Due to rounding, percentages (per row, across columns) may not add up to 100%

**Table 3:** Summary of the trends reported in the scientific literature (systematic review) for scientific knowledge, and for ILK, percentages of population trends by ILK holders and the Trend Index Average. Due to rounding, percentages (per row, across columns) may not add up to 100%

Species	Scientific knowledge	Indigenous and Local Knowledge (ILK)				
		Population trend: difference between current abundance and abundance in ILK holder’ childhood				
	Systematic review	*N*	Decrease	Stable	Increase	Trend index average
	Trend (literature source)		(− 1)	(0)	(1)	
			%	%	%	
Caracal	–	78	2.56	92.31	5.13	0.03
Cheetah	Decrease and locally extinct (IUCN 2017; Willnerd 2018)	71	35.21	63.38	1.41	− 0.34
Jackal	–	80	6.25	87.50	6.25	0
Leopard	Locally extinct (Cabeza et al. 2016b)	77	38.96	61.04	0.00	− 0.39
Lion	Decrease and locally extinct (Dolrenry et al. 2014; Cabeza et al. 2016b; IUCN 2017; Willnerd 2018)	79	79.75	17.72	2.53	− 0.80
Spotted hyaena	Decrease (Willnerd 2018)	76	3.95	77.63	18.42	0.14
Striped hyaena	Decrease (Willnerd 2018)	75	6.67	84.00	9.33	0.03

## Results

### Current abundance according to scientific knowledge and ILK

Jackal and hyaenidae species (including spotted hyaena and/or striped hyaena, see methods) were the most detected species according to the rates obtained from both ecological sampling methods (camera trapping and track survey). With our ecological surveys we did not detect evidence of lion and leopard presence. We identified caracal, jackal and hyaenidae species from the track surveys (see Fig. 1d for an example), whereas we identified cheetah, jackal, spotted hyaena and striped hyaena from the camera-trap photographs (see Fig. 1c for an example; Table 2). Moreover, from the spatial distribution of camera trapping and track survey, we found that jackal and hyaenidae species were widely distributed, whereas cheetah and caracal were limited to one or few locations (Fig. 1a).

The current carnivore abundance according to ILK varied depending on the species under consideration. While there was a high consensus with over 90% of ILK holders reporting caracal, jackal, spotted hyaena and striped hyaena as common (i.e., many individuals seen often, see methods), there was less consensus for cheetah and leopard, with roughly 60% of ILK holders reporting these animals as common. For lion, there was more consensus for low abundances than for high, with 76% of ILK holders reporting it as present but not common (Table 2a). On these same lines, about 53% of ILK holders reported to have seen a lion for the last time more than one year ago, whereas over 70% of the ILK holders reported having seen caracal, jackal, spotted hyaena and striped hyaena that same month (Table 2b).

### Population trend according to scientific knowledge and ILK

We found that cheetah, leopard and lion have a negative trend index average as they were reported as present or common by a higher number of ILK holders in the past (in ILK holders’ childhood) than in the present, whereas we did not find any difference in reports of caracal, jackal, spotted hyaena and striped hyaena, which have a positive trend index average close to 0 (Table 3). For lions, up to 80% of reports revealed a decrease. While there was a high consensus with more than 80% of reports showing that caracal, jackal and striped hyaena were stable, for cheetah and leopard there was less consensus with 63% and 61% of reports indicating that they had remained stable respectively. About 18% of reports showed that spotted hyaenas have increased since ILK holder’ childhood (Table 3).

Despite the lack of scientific ecological studies directly addressing trends in Sibiloi, we gathered information from the systematic review suggesting a general defaunation in the area (i.e., the disappearance of large fauna, Cabeza et al. 2016a, b; IUCN 2017), including carnivore species. However, we found variation depending on the species. Whereas Willnerd (2018) suggests the decrease of lion, cheetah, spotted hyaena and striped hyaena, other literature sources report the local extinction of cheetah (IUCN 2017), leopard (Cabeza et al. 2016b) and lion (Dolrenry et al. 2014; Cabeza et al. 2016b; IUCN 2017). No specific trends of caracal and jackal were found (Table 3).

### Contrast between knowledge systems

Overall, we found points of convergence and divergence between both knowledge systems. On the one hand, by looking at the shared points between both knowledge systems, we differentiated two clear groups that include all species except for the caracal. The first group of species include jackal, spotted hyaena and striped hyaena. For this group, the information gathered suggests they are the most abundant, widely distributed, more frequently and recently sighted species, and with a non-negative trend index average (Fig. 1a; Tables 2, 3). The second group of species include cheetah, leopard and lion. For this group, the information derived from both knowledge systems suggests that they are the less abundant, irregularly distributed, less frequently and recently sighted species, and with decreasing population trends including possible local extinctions (Fig. 1a; Tables 2, 3).

On the other hand, we found points of divergence. First, according to photographic and track rates, abundances are low for all species, whereas high percentages of ILK holders reported them as common. Secondly, for the caracal, while it was only detected in low rates by track surveys (Table 2), 94% of the ILK holders reported it as a common species in the area (Table 2a), and 72% of the ILK holders reported that the last sighting of the species had taken place within the current month (Table 2b). Thirdly, despite the absence of scientific studies addressing trends in Sibiloi, we found a mismatch when complementing the general decline suggested in the literature and the population trends described mostly as stable according to ILK (Table 3).

## Discussion and conclusion

Most efforts in conservation have often focused on convergences between scientific knowledge and ILK that can lead to finding synergies for wildlife management, and have paid less attention to divergences that can indicate stakeholders conflicts and can create challenges in effectively implementing conservation actions (see Miller et al. 2016). While convergences can overlook the perspective of interests of different stakeholders (i.e., preference for carnivore population size), divergences can enhance our understanding of underlying socio-psychological and cultural influences, economic pressures and historical events. Thus, in-depth comprehension of convergences and divergences between scientific knowledge and ILK are of fundamental interest for conservation, policy and practice. Overall, our study brings into focus the importance of acknowledging divergences and exploring them together with convergences. Here we discuss their potential reasons and their implications in conservation. While convergences emphasize the urgency of conservation actions in Sibiloi, divergences highlight existing limitations of our methods and a potential explanation of underlying socio-psychological phenomena for why local people may be willing or not to participate in future conservation initiatives for target species they perceive as abundant. Looking further into both, convergences and divergences, allows us to have an enriched understanding of the carnivore situation and conservation context in Sibiloi.

Both knowledge systems together paint the clearest picture of the status of carnivore species in Sibiloi, with two clearly defined groups of species (except for the caracal). First, three of the studied species (i.e., jackal, spotted hyaena and striped hyaena) that are listed as non-threatened at the global level by the IUCN (Table 1), are found to be the most abundant at the local level (Table 2), widely distributed (Fig. 1a), more frequently and recently sighted by ILK holders (Table 2a, b). These species have also a non-negative trend index average (Table 3). The increasing trend perceived for spotted hyaenas may be due to their extraordinary behavioural flexibility (e.g., excellent hunters and opportunistic scavengers) and high ability to adapt to anthropogenic disturbances, while other species such as striped hyaenas that are primarily scavengers have a moderate ability to adapt to human-dominated landscapes. In addition, because of the differences in social organization, it is easier to see a large number of spotted hyaenas together than for striped hyaenas (see Table 1). Second, cheetah, leopard and lion are listed as threatened globally by the IUCN (Table 1), they are reported to be locally extinct according to the scientific literature and have a negative trend index average (Table 3). They are the less abundant (Table 2), irregularly identified (Fig. 1a), less recently and frequently sighted by the ILK holders in Sibiloi (Tables 2a, b). A local pastoralist said: “Before there were rhinos, zebras, lions, giraffes, leopards and cheetahs. I saw them all when I was a kid, now there are none of these” (Cabeza et al. 2016b). The information derived from both knowledge systems support the implication that there is a need for conservation action regarding these species in the area, especially for lions, leopards and cheetahs. However, it is important to recognise that the preferred carnivore population size may differ between conservation scientists and local pastoralists of Sibiloi who experience the costs of living with these animals. The success of any conservation initiative largely depends on the support of the local communities (Dolrenry et al. 2016). Therefore, it is crucial to partner with the local communities to ascertain their perceptions, attitudes and values towards these species, their potential support for any conservation actions, and the ways in which such initiatives may affect their livelihoods and safety. Simultaneously, conservation initiatives need to take caution to protect the cultural integrity of IPLC without imposing Western assumptions. In this way, conservation approaches that are accepted or, preferably, beneficial to the community may be identified (see Mkonyi et al. 2017 for an example of community-based conservation initiative aimed at reducing livestock depredation by carnivores).

Together with these convergences, our study explores the points of divergence. On the one hand, some mismatches might be explained by the limitations of our methods used (see methods section); from detection failures of both ecological sampling methods (see Torrents-Ticó et al. 2017) to Daasanach misidentifications, as well as insufficient sampling efforts due to extremely low abundance, or not accounting for movement patterns or specific habitat preferences that might affect variation in detectability across species (Burton et al. 2015; Sollmann 2018). These shortcomings could limit the monitoring of wildlife for conservation decisions. However, they could also help to improve sampling design especially for camera trapping and track surveys towards the most appropriate and accurate method for a given species and circumstance. Despite such limitations, some mismatches may have better understating if we compare our track or photographic rates to other studies. For instance, the mismatch for the caracal could be explained because of an insufficient sampling effort if compared to Singh et al. (2014) that required 679.9 trap-nights over four years to spot a single caracal. In addition, if we compare with Gusset and Burgener (2005) (see Table S2), we found that our 0.07 tracks/km is comparable to 0.1 tracks/km. Thus, we could interpret that the Sibiloi caracal population is potentially healthy, as local pastoralists pointed out, and that this species is scarce in nature.

On the other hand, some divergences might be explained by different socio-psychological phenomena. However, it is not easy to understand which socio-psychological phenomena may affect ILK. First, cultural differences could be mediating how people perceive carnivores’ abundances, with local pastoralists and researchers having different culturally-mediated views on when carnivores can be considered as common or present in low numbers (see Camino et al. 2016). For instance, animosity towards spotted hyaenas is common among many African communities, with oral traditions transmitting culturally developed prejudice and lingering antipathy towards the species (Fernández-Llamazares and Cabeza 2018). Although during our interviews, many pastoralists reported an aesthetic appreciation for carnivore species and reported that they are worried that they do not see them anymore, many others stated that every night spotted hyaenas are trying to attack the livestock they are herding. While the scientific community recognizes the ubiquitous nature of such conflicts, it has been generally adamant about the important role of spotted hyaenas as apex predators and scavengers of ecosystems with beneficial contributions to human health and well-being (O’Bryan et al. 2018). Second, high perceptions of risk and damage by local people due to human-carnivore conflicts could be also shaping ILK-based observations of abundances and trends (see Gagnon and Berteaux 2009). In the Daasanach community, livestock has not only an economic value, but also multiple cultural values, which makes livestock losses very difficult to accept. Therefore, carnivore species that have a high impact on the day-by-day pastoralist lives may actually be perceived as being more abundant. Following these lines, those Daasanach pastoralists that herd their livestock for a longer period of time in areas that are largely defaunated of wild herbivores, are in closer contact with carnivore species. Many pastoralists mentioned they need to protect their livestock from these animals that are very dangerous every night. Altogether, our results suggest that ILK should be used with caution, especially when ILK is the only available source of knowledge when focusing on carnivore species abundances and trends (see Caruso et al. 2017). Nevertheless, divergences due to socio-psychological phenomena (e.g., cultural differences, perceptions of risk and damage) should be considered for conservation actions, since this information could help to understand local people’s willingness to reject or embrace conservation initiatives (Manfredo 2008). For instance, according to our field notes, the Daasanach community would be certainly interested in participating in conservation initiatives aimed at reducing human-wildlife conflicts and mitigating damages incurred by carnivore species.

Studies on ILK have contributed to a better understanding of everyday human-carnivore relationships and existing conflicts (e.g., Jhamvar-Shingote and Schuett 2013). Despite ILK contributions, few studies connect ILK with scientific knowledge, and when they do, the reliability of ILK is assessed with scientific knowledge (see Caruso et al. 2017). This is concern-worthy given the vast amount of knowledge-in-use that many communities hold and apply on a daily basis when navigating their close, direct and long-term relationships with carnivores. Therefore, when complementing knowledge systems, no knowledge system should be accepted or rejected unquestioningly; instead, they should be rigorously scrutinized and complemented through respectful and equitable dialogue. In this case, divergences between knowledge systems should not be considered as problematic; rather as an opportunity to further knowledge generation. This study does not rely on scientific knowledge overlooking ILK and it does not defend ILK without questioning it either, here our study adds to and highlights the complementary use of ILK and scientific knowledge, including convergences and divergences to better inform carnivore conservation.

The inclusion of ILK, as a distinct knowledge system, with scientific knowledge into evaluations of carnivores status, trends and potential management actions can cast light on many overlooked interactions between local communities and carnivores, including the communities’ attitudes, values and behaviours, all of which play a critical role in a more inclusive conservation. Following these lines, carnivore conservation can benefit from the Multiple Evidence Base approach (see Tengö et al. 2014) that proposes parallels whereby different knowledge systems are viewed to generate different manifestations of useful and valuable knowledge for better stewardship of our planet. Although the results presented here are case-specific, we consider that our study encourages conservation scientists and practitioners to pay greater attention to ILK, and pair ILK and scientific knowledge to improve our understanding of social-ecological systems, make environmental decisions that are more inclusive, and continue supporting local communities in their pathways towards coexistence.

To conclude, three main recommendations emerge from this complementary study. First, it is important to complement different knowledge systems as independent sources of information to enhance our understanding of the status of carnivore species. Second, the existence of divergences should not be dismissed in social-ecological studies; on the contrary, such discrepancies should be acknowledged and explored together with convergences in order to obtain a more holistic, complete and refined understanding of a given social-ecological challenge. Third, ILK can help us to understand human–carnivore relationships in much more depth, richness and complexity than scientific knowledge alone.

## Electronic supplementary material

Below is the link to the electronic supplementary material.

Supplementary material 1 (PDF 551 kb)
