# Supplementary material for: Convergences and divergences between scientific and Indigenous and Local Knowledge contribute to inform carnivore conservation
Source: Ambio. 2021 Jan 13;50(5):990–1002. doi: 10.1007/s13280-020-01443-4 (PMC8035381; doi:10.1007/s13280-020-01443-4)
Supplement: Supplementary file 1 — Supplementary material 1 (PDF 551 kb) [file 13280_2020_1443_MOESM1_ESM.pdf]

Ambio

Electronic Supplementary Material

This supplementary material has not been peer reviewed.

Title: **Convergences and divergences between scientific and Indigenous and Local Knowledge contribute to inform carnivore conservation**

Authors: Miquel Torrents-Ticó, Álvaro Fernández-Llamazares, Daniel Burgas, Mar Cabeza.

This file includes:

Table S1. Questionnaires used in the semi- structured interviews.

Table S2. Track rates from various study areas across African landscapes.

Table S3. Photographic rates from various study areas across African landscapes.

Table S1. Questionnaires used in the semi- structured interviews. **Village/Area of origin:**

**Coderid:**	**Closest village:**			**Date:**	**Estimated age:**		**Sex:** M or F
	When you were a child, was this animal common/present/absent in Sibiloi?<br><br>Is this animal currently common/present/absent in Sibiloi?			Why do you think that this animal is now more or less abundant?	When and where was the last time that you remember seeing this animal?		Notes
	2=common (many individuals seen often); 1=present in low numbers (some seen occasionally); 0=absent (not seen)			Text	When? (text)	Where? (text)	
**Animal**	Past* (>20 yrs)	Recent* (past 20 yrs)	Now (past year)	*For individuals visibly older than 30 years old, fill the first and last column<br>For individuals visibly younger than 30 years old, fill the second and last column			
Lion							
Leopard							
Cheetah							
Caracal							
Stripped hyena							
Spotted hyena							
Jackal							

Table S2. Track rates from various study areas across African landscapes.

	Tsavo National Park, (Kenya) Henschel et al. 2020	Meibae Community Conservancy (Kenya) Masseloux et al. 2018	Shayamanzi Game Ranch (South Africa) Gusset and Burgener 2005
**Caracal**	-	0.0025	0.1
**Cheetah**	0.04	0.0025	-
**Jackal**	-	0.07	0.73
**Leopard**	0.13	0.01	0.03
**Lion**	0.22	-	-
**Spotted hyaena**	0.83	0.14	-
**Striped hyaena**	0.17	0.002	-

The results from the studies have been transformed to be comparable to our measure based on their method descriptions. The results on relative numbers are still sensible, with the disclaimer that the area is different ecosystems and slightly different transect methodologies.

References

Gusset, M., and N. Burgener. 2005. Estimating larger carnivore numbers from track counts and measurements. *African Journal of Ecology* 43: 320-324.

Henschel, P., L.S. Petracca, S.M. Ferreira, S. Ekwanga, S.D. Ryan, and L.G. Frank. 2020. Census and distribution of large carnivores in the Tsavo national parks, a critical east African wildlife corridor. *African Journal of Ecology* 58: 383-398.

Masseloux, J., C.W. Epps, A. Duarte, D. Schwalm, and M. Wykstra. 2018. Using detection/non-detection surveys and interviews to assess carnivore site use in Kenya. *African Journal of Wildlife Research* 48.

Table S3. Photographic rates from various study areas across African landscapes.

	Rift Valley (Kenya) Schuette et al. 2013	Ngamiland District (Botswana) Rich et al. 2016	Lake Manyara National Park (Tanzania) Steinbeiser et al. 2019
**Caracal**	0.0009	0.014	-
**Cheetah**	0.0008	0.00045	-
**Jackal**	0.005	0.04	0.019
**Leopard**	0.00072	0.05	0.012
**Lion**	0.0035	0.012	0.012
**Spotted hyaena**	0.012	0.097	0.079
**Striped hyaena**	0.015	-	

The results from the studies have been transformed to be comparable to our measure based on their method descriptions. The results on relative numbers are still sensible, with the disclaimer that the area is different ecosystems and slightly different methodologies.

References

Rich, L.N., D.A. Miller, H.S. Robinson, J.W. McNutt, and M.J. Kelly. 2016. Using camera trapping and hierarchical occupancy modelling to evaluate the spatial ecology of an African mammal community. *Journal of Applied Ecology 53*: 1225-1235.

Schuette, P., A.P. Wagner, M.E. Wagner, and S. Creel. 2013. Occupancy patterns and niche partitioning within a diverse carnivore community exposed to anthropogenic pressures. *Biological Conservation* 158: 301-312.

Steinbeiser, C.M., J. Kioko, A. Maresi, R. Kaitilia, and C. Kiffner. 2019. Relative abundance and activity patterns explain method-related differences in mammalian species richness estimates. *Journal of Mammalogy 100*: 192-201.
